# 80 questions for UK biological security

**DOI:** 10.1371/journal.pone.0241190

**Published:** 2021-01-06

**Authors:** Luke Kemp, David C. Aldridge, Olaf Booy, Hilary Bower, Des Browne, Mark Burgmann, Austin Burt, Andrew A. Cunningham, Malcolm Dando, Jaimie T. A. Dick, Christopher Dye, Sam Weiss Evans, Belinda Gallardo, H. Charles J. Godfray, Ian Goodfellow, Simon Gubbins, Lauren A. Holt, Kate E. Jones, Hazem Kandil, Phillip Martin, Mark McCaughan, Caitríona McLeish, Thomas Meany, Kathryn Millett, Sean S. ÓhÉigeartaigh, Nicola J. Patron, Catherine Rhodes, Helen E. Roy, Gorm Shackelford, Derek Smith, Nicola Spence, Helene Steiner, Lalitha S. Sundaram, Silja Voeneky, John R. Walker, Harry Watkins, Simon Whitby, James Wood, William J. Sutherland

**Affiliations:** 1 Biosecurity Research Initiative at St Catharine’s (BioRISC), St Catharine’s College, University of Cambridge, Cambridge, United Kingdom; 2 Centre for the Study of Existential Risk (CSER), University of Cambridge, Cambridge, United Kingdom; 3 Department of Zoology, University of Cambridge, Cambridge, United Kingdom; 4 Great Britain Non-native Species Secretariat, Sand Hutton, Animal and Plant Health Agency, York, United Kingdom; 5 Centre for Wildlife Management, School of Biology, Newcastle University, Newcastle-upon-Tyne, United Kingdom; 6 UK Public Health Rapid Support Team, London School of Hygiene and Tropical Medicine, London, United Kingdom; 7 Centre for Environmental Policy, Imperial College London, London, United Kingdom; 8 UK Department of Life Sciences, Imperial College London, London, United Kingdom; 9 Institute of Zoology, Zoological Society of London, London, United Kingdom; 10 Division of Peace Studies and International Development, University of Bradford, Bradford, United Kingdom; 11 Institute for Global Food Security, School of Biological Sciences, Queen’s University Belfast, Belfast, Northern Ireland, United Kingdom; 12 Oxford Martin School and Department of Zoology, Oxford University, Oxford, United Kingdom; 13 Program on Science, Technology, and Society, Tufts University, Medford, Massachusetts, United States of America; 14 Department of Pathology, Addenbrooke’s Hospital, University of Cambridge, Cambridge, United Kingdom; 15 The Pirbright Institute, Pirbright, Surrey, United Kingdom; 16 Centre for Biodiversity and Environment Research, Department of Genetics, Evolution and Environment, University College London, London, United Kingdom; 17 Marine and Fisheries Division, Department of Agriculture and Rural Affairs Northern Ireland, Downpatrick, United Kingdom; 18 SPRU, University of Sussex, Falmer, Brighton, United Kingdom; 19 OpenCell, London, United Kingdom; 20 Biosecure Ltd, Bourton-on-the-Water, United Kingdom; 21 Earlham Institute, Norwich, United Kingdom; 22 Centre for Ecology & Hydrology, Crowmarsh Gifford, United Kingdom; 23 Centre for Pathogen Evolution, Department of Zoology, University of Cambridge, Cambridge, United Kingdom; 24 Department for Environment, Food and Rural Affairs (Defra), London, United Kingdom; 25 Department for Public International Law, Comparative Law, and Ethics of Law, Law Faculty, Freiburg University, Freiburg, Germany; 26 Arms Control and Disarmament Research Unit, Foreign and Commonwealth Office, London, United Kingdom; 27 Department of Landscape, Arts Tower, University of Sheffield, Sheffield, United Kingdom; 28 Department of Veterinary Medicine, University of Cambridge, Cambridge, United Kingdom; US Army Engineer Research and Development Center, UNITED STATES

## Abstract

Multiple national and international trends and drivers are radically changing what biological security means for the United Kingdom (UK). New technologies present novel opportunities and challenges, and globalisation has created new pathways and increased the speed, volume and routes by which organisms can spread. The *UK Biological Security Strategy* (2018) acknowledges the importance of research on biological security in the UK. Given the breadth of potential research, a targeted agenda identifying the questions most critical to effective and coordinated progress in different disciplines of biological security is required. We used expert elicitation to generate 80 policy-relevant research questions considered by participants to have the greatest impact on UK biological security. Drawing on a collaboratively-developed set of 450 questions, proposed by 41 experts from academia, industry and the UK government (consulting 168 additional experts) we subdivided the final 80 questions into six categories: bioengineering; communication and behaviour; disease threats (including pandemics); governance and policy; invasive alien species; and securing biological materials and securing against misuse. Initially, the questions were ranked through a voting process and then reduced and refined to 80 during a one-day workshop with 35 participants from a variety of disciplines. Consistently emerging themes included: the nature of current and potential biological security threats, the efficacy of existing management actions, and the most appropriate future options. The resulting questions offer a research agenda for biological security in the UK that can assist the targeting of research resources and inform the implementation of the *UK Biological Security Strategy*. These questions include research that could aid with the mitigation of Covid-19, and preparation for the next pandemic. We hope that our structured and rigorous approach to creating a biological security research agenda will be replicated in other countries and regions. The world, not just the UK, is in need of a thoughtful approach to directing biological security research to tackle the emerging issues.

## Introduction

Activities to ensure biological security as a means to protect people, economic interests and the environment, including native biodiversity, are of critical importance to the United Kingdom (UK). The ongoing Covid-19 pandemic had contributed to over 40,000 deaths in the UK and forced a drastic economic slowdown. A recent study has put the potential costs of an unvaccinated influenza pandemic at between £8.4–16.8bn for low fatality scenarios and £55.5–72.3bn for high fatality scenarios [1]. In 2001, an outbreak of foot-and-mouth disease in the UK led to the culling of over six million sheep, cattle, and pigs [2], and economic losses of over £8 billion [3]. The invasive tree disease Ash Dieback is predicted to cost the UK £15 billion over the next one hundred years, with approximately half the cost occurring in the coming decade [4]. The UK Government’s approach to managing biological security threats has evolved with the publication of the *UK Biological Security Strategy* in 2018 [5].

*The Strategy* consists of four pillars: understand current and future risks; prevent risks from threatening the UK and its interests; detect biological risks as early and reliably as possible, and; respond to biological risks to mitigate their impact and speed recovery. These pillars are complemented by two themes: ensuring that government response if underpinned by appropriate scientific capacity and capabilities, and; to capitalise on oppurtunitires offered by the biological sector.

Yet, problems in implementing this strategy can be anticipated due to national and international developments. These include threats from: bioengineering technologies; changes in climate, trade and land-use with unprecedented potential to facilitate the spread of disease agents and invasive alien species; increased speed and volume of international movement of organisms; increased prevalence of antimicrobial-resistant pathogenic organisms and pesticide-resistant arthropod disease vectors, and; deliberate misuse [6]. Tackling some of these new threats, such as the potential for misuse from synthetic biology, may require a drastic shift in how policy usually does risk management [7].

Tackling future biological security challenges within the UK will require evidence-based policy, coupled with rational precaution. One of the key themes of the *UK Biological Security Strategy* is ‘a strong science base’, and it highlights that all actions in biosecurity must be “underpinned by high quality science and evidence if it is to be effective” [5]. This need for evidence-based policy has been acknowledged [8]. Despite this, many biological security topics are neglected and there is a risk that policy-makers will not use evidence to inform decisions, due to lack of awareness or “evidence complacency” [9]. Furthermore, researchers may fail to effectively communicate the nuances of policy-relevant topics.

*The Strategy* is broad and does not identify what research needs to be done to ensure each pillar is met. Nor does it specify the research needed to ensure actions are robust to future developments in biosecurity. Indeed, t the best of our knowledge no such list of priority, tractable and unanswered rearch questions exist. We seek to remedy this by providing a targeted research agenda which meets both of these needs. To identify the most pressing questions for UK biological security, we used structured expert elicitation to generate a set of research questions of relevance to policy makers and UK biological security. When paried with the objectives and framework of the *the Strategy*, this constitutes a well-grounded research agenda for biosecurity in the UK.

Many emerging biosecurity dilemmas, such as the malicious use of synthetic biology, require new approaches to biosecurity, including the engagement of social scientists and policy-makers in forecasting [10]. This is a step in this direction by having a diverse group of scholars, practicioners and policy-makers collaboratively contemplate the most pressing current and future challenges in biosecurity.

This approach draws on a series of similar studies in fields including global agriculture [11], global conservation biology [12], research priorities for poverty prevention and reduction [13], the global post-2015 development agenda [14], invasive species [15] and ecological questions of high policy relevance in the UK [16].

Expert elicitations of research priorities have real-world impact. For example, an exercise to determine priority questions for research in the Antarctic and Southern Ocean [17] has had a significant impact on policy, research practice and funding [18]. Moreover, previous studies have helped to direct research efforts and funding allocations. This is the first time that we are aware of such an exercise being performed for the biological security agenda in any nation.

We used a broad definition of the term ‘biological security’ in the development of these research questions, in line with the definition outlined in the *UK Biological Security Strategy*. That is, the protection of population and economic interests from naturally arising, accidentally occurring or deliberately caused biological risks (particularly significant disease outbreaks). This covers biological risks affecting people, the economy and the environment—whether these arise naturally, from deliberate actions, from unintended release or from the unanticipated consequences of deliberate release [5]. It is worth noting that biological security has more specific meanings within several of the disciplines and sectors represented by the workshop participants.

## Methods

**Fig 1:**
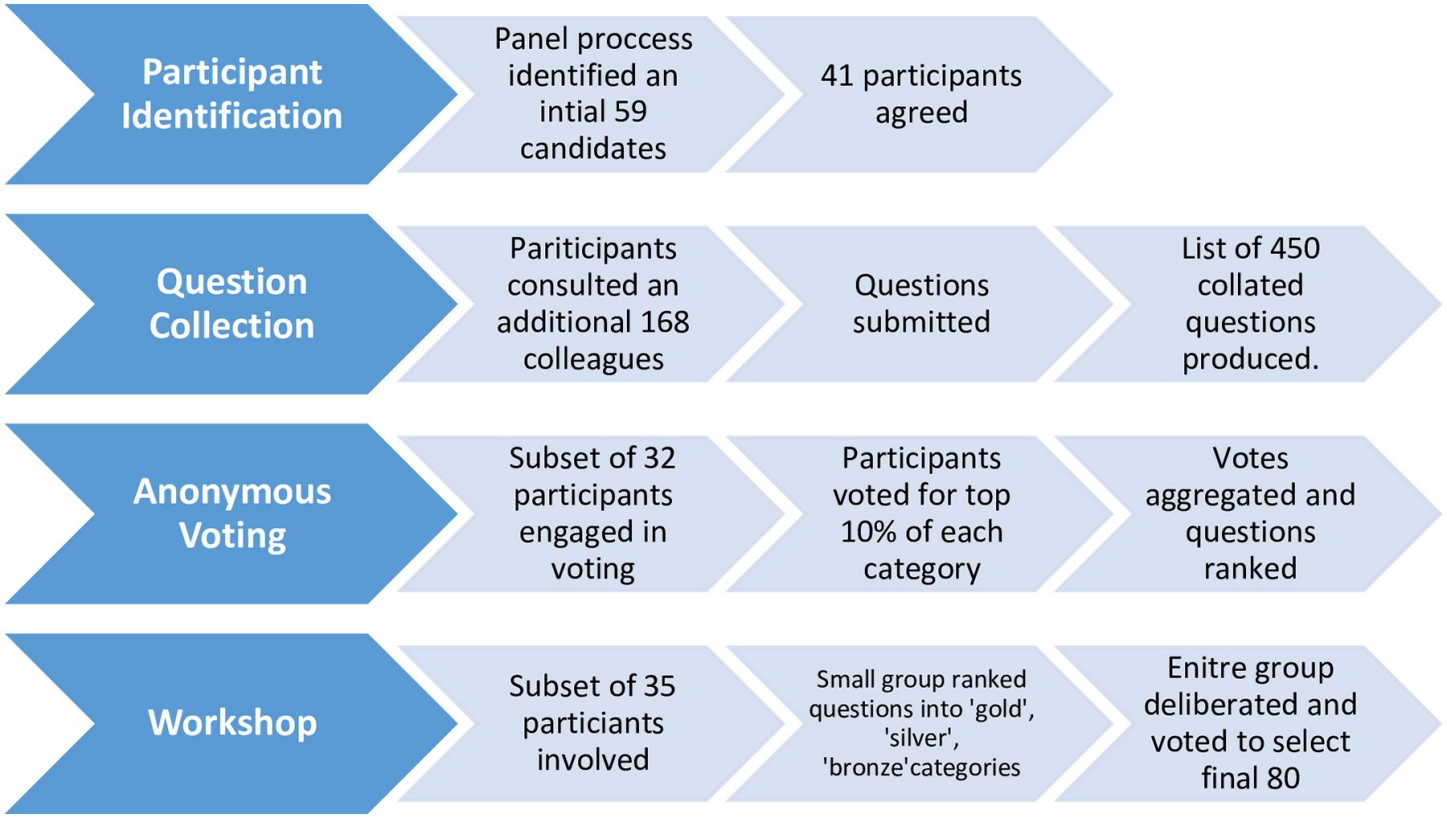
An overview of the 80 questions process.

Producing the 80 questions involved a four-phase process of structured expert elicitation. Fig 1 provides an overview of the approach. Phase 1 involved using an expert panel to identify and contact a group of 59 experts from a range of relevant disciplines. 41 agreed to join the process. In phase 2 these participants were asked to draw on their networks to propose tractable, unanswered research questions. This resulted in a list of 450 collated questions split into 6 categories. Phase 3 centreeed on anonymous voting by a subset of 32 participants who selected the top 10% of each category. The voting results were used to inform discussion during a one-day workshop on 17 July 2019 at the University of Cambridge. A subset of 35 participants attended the workshop and through a process of deliberation, voting and ranking, refined the questions and selected the top 80.

A panel of six, with experts (Catherine Rhodes, William Sutherland, Lalitha Sundaram, David Aldridge, Des Browne and Sean ÓhÉigeartaigh) across different areas of biological security, was convened to identify relevant topics and participants for the exercise. Expert identification is critical in ensuring an adequate breadth of knowledge. The panel focused on eight key topics for biological security: bioengineering and novel technologies, dual use concerns, media, plant disease, zoonoses, international policy and law; invasive species; and pandemics and epidemiology.

On the 6^th^ of April, 2019, invitations were sent to 59 experts from a range of different organisations. This resulted in a group of 41 participants from 12 universities, 5 government agencies (Defra, the Foreign and Commonwealth Office, the Non-Native Species Secretariat, the Defence Science and Technology Laboratory, and the Department of Agriculture, Environment and Rural Affairs (fisheries) in Northern Ireland), 5 other institutes, one business representative and a parliamentarian (see S1 Appendix for a list of participants and their affiliations). Henceforth, ‘participants’ refers to any member of this group of 41, including the subset of 35 participants who attended the workshop. The exercise intentionally gathered experts from across science, policy and practice. This was done such that policy-makers could screen questions for their relevance and significance, while researchers provided the expertise to both pose insightful questions (that could be addressed by a research programme) and help filter out questions that have already been answered in the literature. This has the added benefit of helping foster relationships across these domains, a measure that Evans et al have called for to combat evidence complacency [19].

Each participant was asked to submit between 5–25 questions after consulting with relevant colleagues in their wider networks. Questions had to be: be answerable through a realistic research design; have a factual answer that does not depend on value judgments; and be at an appropriate spatial and temporal scale and scope. Participants were instructed to make sure that questions related to impacts and interventions, contained a subject, an intervention, and a measurable outcome.

The questions were collated in the six categories with overlapping questions merged, resulting in an initial set of 450 questions (provided in S2 Appendix). This intial list is contained in S2 Appendix. The participants consulted an additional 168 experts in formulating these questions. The panel did not actively screen who the participants consulted. Instead this was left to participant discretion by asking them to draw on networks and colleagues that they considered relevant to the exercise. 60 of these wider experts provided direct questions that were part of the initial list of 450. The rest provided advice and comments more broadly, including on voting, and areas to examine or avoid. The categories were loosely based on the topics used by the selection panel to identify participants, with adjustments to suit the range and number of questions. The aim of the categorisation was to provide a logical method to coalesce questions into roughly equal groups for experts to focus on. Naturally, there are significant areas of overlap and connection, and many questions cut across multiple groupings. (The categories were revised for the final list of questions to better reflect the composition of selected questions. This categorisation was agreed to by consensus of participants during the process of drafting).

The list of 450 questions was then sent back to participants for voting on the 24^th^ of June, 2019. They were asked to indicate their top 10% for any area in which they had sufficient expertise. Seven participants voted in one area, nine in two, four in three, two in four, two in five, and eight in six. This resulted in a total of 32 votes (several participants were not able to engage in the voting process). The votes were then compiled and used to rank the questions. The ranked list was sent back to respondents in advance of the workshop on Tuesday the 16^th^ of July, 2019.

The workshop involved 35 participants (six could not attend), see S1 Appendix for further details. Each of the sessions was facilitated by a topic expert and consensus was sought on each question (including cutting, refining and ranking). Majority voting was not used until the final plenary session when refining the penultimate list into the final selection of 80. While the outcome reflects a consensus view, some participants may not endorse the inclusion of some questions.

Each of the facilitators was from the initial panel of six experts who created the original participant invitiation list. They conducted the facilitation by first restating the initial question on the shortlist, then asking for clarifications or amendements, then asking how the questions should be ranked. The last point involved placing questions into categories of gold, silver and bronze. Gold questions were defined as “suitable, compelling and likely to make it into the final 100.” Silver questions were designated as “generally suitable and have a decent potential to make it into the final 100.” Bronze questions were described as “lower quality but still show the promise to be advanced into the final 100.”

The morning sessions of the workshop had small groups working in parallel explicitly on the different categories. This was completed over two sessions to reduce the number of questions and improve the formulation of those that remained. The afternoon sessions concentrated on further refining and reducing the questions while ranking them into categories of gold, silver and bronze. These sessions were conducted across two clusters of topics: one including bioengineering, securing against misuse, and policy and law; and the other comprising invasives, pandemics, plant disease and zoonoses. The penultimate list was composed of 80 gold, 47 silver and 46 bronze questions. This list was then further sharpened during a plenary session. One gold question was dropped to bronze and 10 to silver. 8 silver questions were promoted to gold, as well as 3 bronze, resulting in the final list of 80 gold questions. The final list was then regrouped into a more appropriate split of topics across: bioengineering, communication and behaviour change, disease threats, invasive alien species, policy and governance and securing against misuse.

Our expert elicitation did not involve human subjects, but rather expert contributors (the co-authors). Thus, ethics committee approval was not sought as is common-practice for similar Delphi exercises. Contributors were informed and agreed with the intent to publish at the outset, the nature of the structured expert elicitation process and were aware that they could withdraw at any time. This information was communicated, and informed consent achieved, via email.

## Results

The resulting questions are grouped into six categories. Although the categories used are one reasonable way of grouping the questions, their boundaries sometimes overlap and many questions cover multiple, inter-related topics. The categories are, however, a useful way of clustering the questions and identifying patterns, but they should not be regarded as a definitive typology.

**Fig 2:**
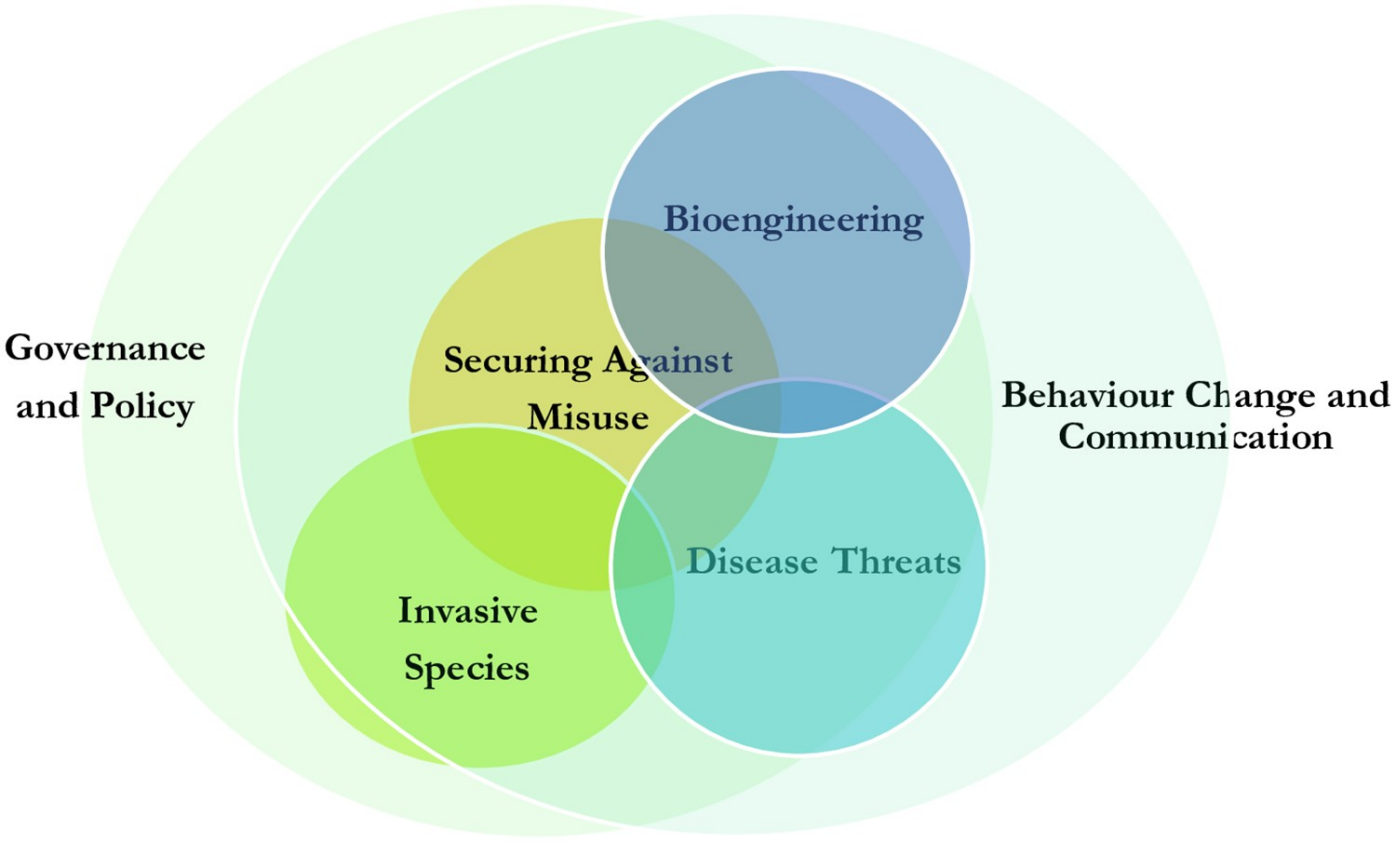
A venn diagram of the 80 questions categories.

The questions are arranged into six interconnected categories. These categoriges have important linkages and overlap, as displayed in Fig 2. These links underpin the need for both an interdisciplinary approach, such as the one taken here, and for biosecurity policy which cuts across topics. Most notably, governance and policy, as well as communication and behaviour change, encompass each of the more distinct biosecurity areas of disease threats, bioengineering, invasive species and misuse. Both *Securing Against Misuse* and *Bioengineering* are two topics that have substantial overlap in thinking about the dual-use implications of emerging technologies.

### Bioengineering

Rapid changes in bioengineering technologies present both biosecurity threats as well as the possibility of new tools to address these challenges. We take a broad definition of bioengineering as “the application of ideas, principles and techniques to the engineering of biological systems” [20]. CRISPR/Cas mediated gene silencing and gene editing (and related technologies) have the potential to modify existing pathogenic organisms and resulting toxins—or to create new ones—in ways which might advance understanding of and response to disease threats, but which might also facilitate misuse or result in increased toxicity and/or virulence. New means of sharing information covertly, such as the ‘dark web’ (Tor anonymity network), have opened up additional acquisition pathways [21] which could be put to malicious use by hostile individuals or groups. Furthermore, new technologies may require new policies, such as the harmonisation of regulations on synthetic biology [22]. Given the uncertainty of future biotechnological capabilities [23], the questions in this section are forward-looking. They also incorporate a broad range of different topics in biotechnology, including challenges that stem from the convergence of biotechnology with other technologies such as cyber-biosecurity (encompass[ing] cybersecurity, cyber-physical security and biological security as applied to biological and biomedical-based systems) [24] threats, synthetic biology and de-extinction [24]. The questions cover the challenges and opportunities posed by new technologies (questions 1–3) and the mitigation options for managing emerging technologies (questions 4–6).

1 Do advances in custom DNA synthesis technologies, and their widespread availability, pose biological security threats to the UK from human-engineered agents, and how can these threats be mitigated or prevented?
2 What new biological security challenges and opportunities are posed by new genetic codes?
3 What are the major pathways by which a biologically engineered threat could enter the UK?
4 How could international import and export regimes be improved to respond better to the complexities of synthetic biology and other new and emerging biotechnologies?
5 What analysis is required to fully scope cyber-biosecurity challenges and responses?
6 What principles and policies (if any) should the UK adopt in relation to de-novo species, such as those created with de-extinction technologies?

### Communication and behaviour change

It has long been understood that simply informing people that something poses a risk does little to convince them to change their behaviour [25]. The adoption of, and compliance with, biological security practices is a critical, yet understudied, subject [26]. Some issues have attracted increasing attention, including the role of social media in amplifying the perception of risk [27] the impact of governance arrangements [28] and the opinions of farmers on biological security measures, with case studies conducted in the UK [29], Denmark [30], Spain [31] and elsewhere within Europe [32, 33]. The questions in this section build on this platform to explore new topics. They are as much about the need to determine what is a valid security concern as they are about proposing new ones. These questions are addressed to a wide audience, from farmers to scientists, policy-makers and members of civil society. The focus spans the organisational, social and technical aspects of risk. The questions range from assessing the current state of public perception and policy (questions 7–8) to determining the most effective communication and behavioural change actions (questions 9–14) [34, 35], and how these can be implemented into fair and effective processes (15–17).

7 How does public perception of biological security risks in the UK compare with expert assessment?
8 How do codes of conduct influence individual and group behaviour with regard to biological security?
9 What are the most effective and/or efficient methods for raising awareness and changing behaviours in favour of better biological security practices?
10 What are the most effective methods to communicate risk of a disease in the UK where name recognition might be greater for diseases unlikely to occur (cholera, Ebola) than diseases most likely to occur (campylobacteriosis)?
11 How can biological security and biosafety risks best be understood, evaluated and communicated in partnership with different audiences?
12 What steps are required to ensure that appropriate awareness-raising, oversight, and professional training are in place to promote a biological security culture of responsibility amongst practitioners?
13 How do we best effect human behaviour change to enhance biological security?
14 How do we communicate and share knowledge with farmers and other animal keepers to develop and apply best biological security advice, especially during periods when epidemic risk is low?
15 How can expert opinion and scientific evidence be used systematically to inform and shape biological security policies?
16 How can we better incorporate biological security topics in the curricula of the training of natural and social scientists?
17 How do we reflect different interests and capture a broad range of voices to clarify perceived risks and weigh the benefits against the risks?

### Disease threats

Diseases are potentially severe threats to biological security. These include new diseases that spread from animals to humans, new plant and animal diseases, existing human, plant and animal diseases that spread into new areas, pathogenic agents that become resistant to existing treatments (e.g., drug-resistant *Mycobacterium tuberculosis*), diseases that continue to have severe impacts on people despite existing treatments (e.g., HIV-AIDS), and diseases that change in severity when introduced into new settings (e.g., amphibian chytridiomycosis). There were several questions about the impacts that such diseases could have (questions 18–21) and the potential shifts that could occur due to changes in climate, land use and disease vectors (questions 22–24) [36–38], or genotypic or phenotypic changes in influenza viruses (questions 25–27). Influenza virus was the focus of several questions as its potential to change virus genotypes and phenotypes, as well as its mode and speed of spread makes it a serious pandemic threat. There were also several questions about disease surveillance, the possibility of using new technologies for detection (questions 28–31), and methods of preventing or managing disease outbreaks (questions 32–35), including methods of developing new technologies for managing outbreaks. Unless specifically stated otherwise, these questions refer to diseases of people, wild and domesticated animals and plants. We expect that the ongong Covid-19 pandemic will significantly impact research priorities. Many of the questions outline here, specifically on disease surveillance, detection and quarantine, will be salient and of immediate use to existing efforts to mitigate Covid-19. Others, such as questions relating to an influenza pandemic, may well help the uK prepare for the next major disease outbreak.

18 How pathogenic to humans are the various predicted pandemic influenza viruses?
19 What are the human health and wellbeing impacts of agricultural and wild plant and animal pathogen outbreaks?
20 What are the economic, societal and biodiversity impacts of wildlife diseases in the UK?
21 What are the consequences of failing to contain the rise of antimicrobial resistant infections and what are the best mitigation methods?
22 How does changing land use affect the spread and impact of pathogens?
23 What disease risks are associated with the changing distribution and ecology of arthropod disease vectors?
24 What new or increased pathogen and pest threats will the UK face in a world changed by different climate change and human development pathway scenarios?
25 What are the within-host evolutionary pathways for avian influenza viruses to evolve to transmit amongst humans, and the probability of their occurence?
26 Of the phenotypic changes needed for a non-human influenza virus to adapt into a pandemic virus in humans, which ones already exist in circulating non-human viruses worldwide?
27 What amino acid substitutions are necessary to make avian non-human influenza viruses in different subtypes and genetic lineages transmissible among humans and cause an influenza pandemic?
28 What technological and other approaches can improve wild and agricultural plant and animal disease surveillance in the UK?
29 How can new technologies be used in syndromic surveillance systems, including detection thresholds, for disease threats in the UK, to more effectively identify unusual events and reduce time to alert?
30 What tools could most effectively improve the detection of slowly-spreading epidemics, such as HIV, vCJD or cancer-causing pathogens such as HPV?
31 To what extent can social media platforms be usefully integrated into early detection/warning systems in the UK?
32 What approaches are possible to protect against currently unknown emerging infectious diseases and how cost-effective might they be?
33 How can we better understand and model human behaviour during disease outbreaks, including deliberate release events?
34 How do we best mobilise the human resources required in health, and veterinary and vector control, services at the speed necessary to treat human patients and control a serious outbreak?
35 If human quarantine were required, how could it be effectively implemented and which actions would most effectively achieve compliance?

### Governance and policy

Biological security governance and policy is continually challenged by the changing nature of biological risks and by the cross-sector nature of many of those risks. We define governance broadly as the set of public and private institutions, norms, regulations and decision-making procedures that determine action in certain issue areas [39]. Policy is the development and implementation of laws, regulations and other instruments by a governmental entity to create or maintain societal change. It also must achieve a balance between management of risks and facilitation of benefits, particularly as the bio-economy (the production, utilization and conservation of biological resources to provide information, products, processes and services) [40] increases in size and significance in the UK [41, 42].

Effective coordination across local and national government in the UK is vital for biological security [5]. The nature of threats to biological security necessitates a focus beyond the national level in policy and governance, and coordination with many other national, regional and international organisations [43]. One of the main forums in which the UK is consistently engaged is the Convention on the Prohibition of the Development, Production and Stockpiling of Bacteriological (Biological) and Toxin Weapons and on Their Destruction (BTWC) and the related Meetings of Experts, States Parties and Review Conferences which it has made numerous submissions to [44].

There is also increasing recognition that biological security governance needs a combination of top-down and bottom-up approaches–incorporating engagement with a range of stakeholders including scientific and technological communities, agricultural, land management and conservation groups, and the public.

Many of the questions in this section are directed towards better understanding UK capacities for coordination of, and engagement in, biological security governance, across the stages of understanding and responding to threats and vulnerabilities, and monitoring and evaluating the effectiveness of existing approaches. Some are of wider importance and have already been discussed internationally, including how to manage ‘information hazards’—the dissemination of data and knowledge that may cause or enable harm—arising from bioengineering research [45]. The questions encompass understanding current challenges and threats (questions 36–42), examining and creating different potential options and approaches (questions 43–50), and policy specifically for research and information hazards (questions 51–52).

36 How do UK overseas territories experience and create different biological security threats to the UK?
37 How might different understandings of the term ‘biological security’ be problematic for coordinated action across the UK government and for effective engagement with expert communities?
38 What have been the barriers to success of the different UK biological security regimes (e.g. wild and domestic animal health, plant health, aquatic health, bee health, invasive non-native species)?
39 Which components of the food system in the UK are most vulnerable to biological security risks?
40 What infrastructure (e.g. transport, health, and power) is most vulnerable to biological security threats?
41 What are the key challenges and opportunities that Brexit poses for UK biological security, and how can these best be responded to?
42 What are the most significant re-emergent biological security threats and do our current threat characteristics accommodate them?
43 What could be done to accelerate investment and development of novel, agile, low-cost systems that could be used for distribution and responsive manufacturing of therapies to combat infectious human, animal and plant disease outbreaks?
44 How can we best build capacity in Parliament to help implement the *UK Biological Security Strategy*?
45 How effective are mechanisms to support cross-government coordination necessary for implementation of the *UK Biological Security Strategy*?
46 How effective are the methods being used to monitor the implementation of the *UK Biological Security Strategy*?
47 How can natural capital valuation help prioritize biological security efforts?
48 How can new biological, computational and hardware technologies be used to balance border controls and trade in living materials such as seeds, plants, livestock and wild animals, while reducing the risk of pathogen transmission?
49 How can we measure the success and failure of biological security interventions in the UK and adaptively use this to improve our responses?
50 What mechanisms exist for recognizing and dealing with the unknowns in biological security?
51 How do we design measures to improve biological security that do not unnecessarily restrict scientists from conducting fundamental and applied research?
52 How do we manage ‘information hazards’?

### Invasive alien (non-native) species

Invasive Alien Species (IAS) are widely considered to be one of the leading drivers of biodiversity loss [46] and are estimated to cost the UK economy at least £1.7 billion annually [47]. Furthermore, invasive parasites and pathogens, which may be introduced with alien host species, are known to threaten human health, native wildlife, fisheries, forestry and food security [48]. We use IAS to encompass non-native animals, plants and parasites/pathogens. The number of alien species establishing in the UK and globally is increasing [49, 50], in tandem with increasing globalization and associated trade [51, 52]. Expansion of transport networks, advances in technology, global environmental change, and geopolitical factors are introducing new risks, vectors and pathways [48]. Successful management of IAS in the UK and Overseas Territories requires effective horizon-scanning programmes [46], risk analysis [53, 54], well-directed surveillance and monitoring schemes, and evidence-based response plans [15, 55, 56]. The questions range from investigating the transport, introduction, establishment and spread of IAS (questions 53–57), to analysing and predicting potential impacts (questions 58–60), assessing existing and past actions (questions 61–63), and developing effective management practices (questions 64–67).

53 What are the major and emerging vectors and pathways, such as changing trade patterns, by which invasive alien species could enter or spread within the UK?
54 What are the current propagule/colonisation pressure patterns of invasive alien species in the UK?
55 What are the biological security risks from importing soil (including rooted plants)?
56 Are there differences in range shifts under climate change between native and invasive alien species?
57 How can climate change mitigation policies (e.g. water transfers, changes in crops, biofuels, re-forestation) promote or retard invasive alien species?
58 What is the invasion debt (i.e. established invasive alien species yet to exert impact) in the UK and how does this vary regionally?
59 What are the social/cultural impacts of invasive alien species?
60 What risk do invasive animal and plant species pose in terms of parasite/pathogen introduction and transmission?
61 How effective has biological security been in preventing biological invasions in other countries and how can we translate effective practice to a UK context?
62 What has been the success of horizon scanning activities for predicting and managing invasive alien species?
63 How well are the UK Overseas Territories managing the impact of invasive alien species and controlling the risks of further invasive alien species?
64 How can citizen science be improved as a method of surveillance and monitoring invasive alien species?
65 What new policy options are required to reduce risks from the widespread distribution of organisms via internet sales?
66 What determines the optimal allocation of resources in (i) preventing entry, (ii) early identification and eradication, (iii) controlling and (iv) mitigating impacts of invasive alien species?
67 Which management practices for invasive alien species (e.g. culling, quarantine, chemical control, biological control, genetic control) are most acceptable to different groups of stakeholders?

### Securing against misuse

Biological research and associated technologies have the potential to be deliberately misused. Historically, several countries, including the UK (until the late 1950s), have had offensive biological warfare programmes and there have been occasional instances of bio-terrorism by non-state actors [57–61]. The long-standing international prohibition on the development and use of biological weapons, embodied in the BTWC that entered into force in 1975, continues to have a high level of support with 183 States Parties [62]. There are acknowledged weaknesses in the BTWC regime. The BTWC has no meaningful verification measures such as declarations or on-site inspections. An attempt to develop these and other associated measures collapsed in 2001 when the US rejected a draft protocol that emerged from the Ad Hoc Group negotiations. Given this gap, and the fact that many BTWC States Parties do not have effective national implementation mesures in place, action against misuse continues to be necessary. This is all the more important in the context of scientific and technological advances that may make destructive capabilities available to a wide range of actors. Such developments include access to table top DNA synthesis, cheaper and easier genetic engineering [63], an increased reliance on data and digital infrastructure, and growing vulnerability to cyber-biological security attacks [64]. A robust assessment of biological security should consider rapidly-changing scientific and technological landscapes [65], types of actors and access, as well as procedures to recognise and mitigate deliberate attacks.

Questions in this section cover: drivers and enablers of misuse and potential responses to them; technological advances that can assist in securing against misuse; principles and processes that can inform the balancing of benefits and risks of research and innovation; and how to achieve appropriate and effective governance, with a focus on how dual use research is managed in the UK [66]. The questions address the changing nature of misuse (questions 68–71), the effectiveness of mitigation options (questions 72–75), and the broader governance frameworks for managing misuse (questions 76–80).

68 What is the most plausible way for a range of non-state actors to generate a biological threat in the UK?
69 How could new technologies aid monitoring and verification of biological weapons development, production, and use?
70 What are the implications of intangible technology, such as tacit knowledge and the accessibility of the knowledge base, for enabling misuse?
71 What are the driving forces likely to lead to the use of biological and toxin weapons in the coming decades (e.g. advances in science and technology, the changing nature of warfare and the decline in the use of multilateral agreements), and how might these forces best be countered?
72 How do we assess the impact of the development of different intersecting technologies the potential for misuse?
73 What are the most effective interventions that the UK can take to mitigate the dark web becoming a technological vector for biological security threats?
74 What new means might be developed to make the operations of the BTWC more effective, and how can the impact of these changes be assessed?
75 How effective, transparent and timely are the mechanisms in the UK for monitoring and controlling research with potential for dual use?
76 What types of governance measures are most appropriate at different stages of technology development (from initial research through to product)?
77 What constitutes dual use research in the UK, how do we determine it, and who decides?
78 What guiding principles would be required to balance the benefits of scientific research with the responsibility to prohibit and prevent the development and acquisition of biological weapons?
79 What methods are effective in engaging wider communities when the research proposed or being conducted raises significant dual use issues? Do these methods differ between public and private settings?
80 What types of proportionate and adaptive governance systems, and assessment procedures, need to be in place to address dual use research concerns and ensure a balance between benefits and risks?

## Discussion

These 80 questions constitute a robust research agenda in response to the *UK Biological Security Strategy* (2018). The questions in each topic can be clustered into understanding the nature of the threats—including their spread and impacts, assessing existing actions, and exploring the most effective management and mitigation options. They largely focus on better comprehending the problems and mapping out the most effective solutions. The categories covers the spectrum of biological threats identified by the *Strategy*—natural, accidental, and deliberate—and cut across its four pillars. This makes both these focal areas, and the important, neglected research questions under each, a effective guide for implementing the *Strategy*.

The questions generated by this process are tailored to the UK and reflect the consensus view of the participants, but many may also align with priorities in other countries, especially within Europe, and will clearly have broader importance beyond the UK. Importanlty, the process used to create the questions is generlaisable. We hope that this structured, rigorous approach to creating a research agenda for biological security will be replicated by other countries and regions. The world is in need of a thoughtful and directed approach to biological security research to meet the challenges of the twenty-first century. This is a national proof-of-concept for such an approach.

While the exercise generated consensus around a set of impactful, tractable questions, it has some limitations. First, the exercise is limited by the initial set of participants chosen and the expertise found in their networks. For example, there were no participants specialising in laboratory safety or in antibiotic resistance, although several had some experience in these areas. Third, participants in such exercises often prefer general over specific questions [16]. Finally, we have not presented the research questions in the context of a particular theory of risk assessment and response, such as Utility theory. Although this could emerge while addressing several of the questions listed above (e.g., those concerning Communication and Behaviour Change). However, we did intentionally target participants from across a diverse set of disciplinary and occupational backgrounds. Despite some gaps, we are confident that if the process was replicated with a comparably diverse group, similar results would be obtained. The inclusion of a variety of scholars from both physical and social sciences was intentional and imperative. This is because co-evolution of these perspectives has been critical for relevant areas such as synthetic biology [67].

Second, the exercise did not include any specific measures to discover highly neglected yet more impactful questions. Goodwin and Wright [68] analysed a range of forecasting methods and found that none sufficiently incorporated measures to anticipate low-probability, high-impact events. They suggest incorporating intentional devil’s advocates (intentionally and directly challenging a predominant view for the sake of debate) to overcome frame blindness—ovesights in common ways of viewing and predicting problems—and create more radical and unforeseen ideas. A future version of a biological security questions workshop could use such recommendations to better elaborate questions concerning low-probability, high-impact events.

Steps were taken to mitigate these limitations. The effect of individual biases was minimised by our panel-based selection process that targeted a large set of participants from a broad range of disciplines, from practitioners to policy-makers. Moreover, we were aware of the attraction towards generic questions, and the facilitators therefore directed the group to work towards an appropriate mix of general and specific.

The process of generating policy relevant questions through structured expert elicitation provides one promising avenue to build connections across the science-policy interface, and develop a relevant, targeted evidence base. We believe that this process can be successfully repeated for other geographical areas, and more refined topics, such as biological security interventions in bioengineering. We would also welcome a further replication of this study in the UK context. This could help to identify new, emerging priorities, particularly in the wake of Covid-19. It is a method capable of informing policy and research across the tumult of rapidly changing risks, opportunities and politics in biological security.

Addressing these 80 questions has the potential to provide a comprehensive evidence base on biological security threats and management actions in the UK, assisting achievement of the objectives of the *UK Biological Security Strategy*. This exercise and the resulting questions provide the foundation for co-production between policy-makers, practitioners and scholars, as well as a guide for funders and donors to assess their investments in the biological security agenda.

## Supporting information

S1 Appendix Supplementary materials. (DOCX)

S2 Appendix (DOCX)
