# Supplementary material for: 80 questions for UK biological security
Source: PLoS One. 2021 Jan 6;16(1):e0241190. doi: 10.1371/journal.pone.0241190 (PMC7787535; doi:10.1371/journal.pone.0241190)
Supplement: S1 Appendix — (DOCX) [file pone.0241190.s001.docx]

S1 Appendix: Supplementary Materials

Below is a list of all of the participants in the exercise, including their affiliated institution and in which stages they participated.

| **Contributor** | **Institution** | **Participation** | **Consulted** |
| --- | --- | --- | --- |
| David C. Aldridge | Department of Zoology, University of Cambridge (Academic). | Participated in all stages. | 2 |
| Olaf Booy | Great Britain Non-native Species Secretariat, Sand Hutton, Animal and Plant Health Agency (Government).  Centre for Wildlife Management, School of Biology, Newcastle University (Academic). | Participated in all stages. | 9 (from the GB Non-native Species Risk Analysis Panel and Newcastle University). |
| Hilary Bower | UK Public Health Rapid Support Team, Faculty of Epidemiology and Population Health, London School of Hygiene and Tropical Medicine (Academic). | Participated in all stages. | 8: John Edmunds (provided questions); David L. Heymann (provided questions); Emilio Hornsey (provided questions); Punam Mangtani (provided questions); Punam Mangtani (provided questions); Dilys Morgan (provided questions); Olivier le Polain (reviewed questions); and Jimmy Whitworth (provided questions). |
| Des Browne | House of Lords member (Government).  Centre for the Study of Existential Risk, Cambridge University (Academic). | Provided issues. | 0 |
| Austin Burt | Faculty of Natural Sciences, Department of Life Sciences, Imperial College London (Academic). | Provided questions. | 0 |
| Mark Burgmann | Centre for Environmental Policy, Imperial College London (Academic). | Provided questions, voted and helped with drafting. | 5. All biological security specialists with backgrounds in Australian biological security |
| Jason Chin | Centre for Chemical and Synthetic Biology, University of Cambridge (Academic). | Provided questions. | 0 |
| Andrew A. Cunningham | Institute of Zoology, Zoological Society of London (Academic and NGO). | Participated in all stages. | 0 |
| Malcolm Dando | Division of Peace Studies and International Development, University of Bradford (Academic). | Participated in all stages. | 3 |
| Jaimie T.A.Dick | Institute for Global Food Security, School of Biological Sciences, Queen’s University (Academic). | Participated in all stages. | 2. A PhD student (biological security expertise) and a retired Professor of Biological security (Joe Caffrey). |
| Christopher Dye | Oxford Martin School & Department of Zoology, Oxford University (Academic). | Provided issues, helped with drafting. | 0 |
| Belinda Gallardo | Department of Zoology, University of Cambridge (Academic). | Participated in all stages. | 6 (comments were received from 2). |
| Charles J. Godfray | Oxford Martin School & Department of Zoology, Oxford University (Academic). | Participated in all stages. | 3 |
| Ian Goodfellow | Wellcome Trust - Cambridge Centre for Global Health Research, Cambridge University (Academic). | Participated in all stages. | 0 |
| Simon Gubbins | The Pirbright Institute (NGO). | Paraticipated in all stages. | 43: 36 by email, 7 in-person. 5 provided questions. |
| Lauren A. Holt | Centre for the Study of Existential Risk, Cambridge University (Academic). | Participated in all stages. | 1 |
| Kate E. Jones | Institute of Zoology, Zoological Society of London (NGO).  Centre for Biodiversity and Environment Research, Department of Genetics, Evolution and Environment, University College London (Academic). | Participated in all stages. | 6 (with comments from 2). |
| Hazem Kandil | Biological security Research Initiative at St Catharine’s (BioRISC), St Catharine’s College, University of Cambridge (Academic). | Participated in all stages. | 0 |
| Luke Kemp | Centre for the Study of Existential Risk, Cambridge University (Academic). | Organiser. Participated in all stages. | 2 |
| Mairi Kilkenny | Biological security Research Initiative at St Catharine’s (BioRISC), St Catharine’s College,University of Cambridge (Academic). | Provided questions. | 0 |
| Phillip Martin | Department of Zoology, University of Cambridge (Academic). | Participated in all stages. | 0 |
| Mark McCaughan | DAERA Northern Ireland Fisheries (Government). | Provided questions and contributed to drafting. | 0 |
| Caitríona McLeish | Harvard Sussex Program on Chemical and Biological Weapons, SPRU University of Sussex (Academic). | Participated in all stages. | 1 |
| Lorna Miller | Defence Science and Technology Laboratory. | Provided questions, voted and participated in workshop. | 0 |
| Kathryn Millett | Biosecure (NGO). | Participated in all stages. | 2 |
| Sean S. ÓhÉigeartaigh | Centre for the Study of Existential Risk, Cambridge University (Academic). | Participated in all stages. | 0 |
| Nicola J. Patron | Earlham Institute (NGO). | Participated in all stages. | 10 |
| Helen E. Roy | NERC, Centre for Ecology & Hydrology, Lancaster University (Academic). | Participated in all stages. | 0 |
| Catherine Rhodes | Centre for the Study of Existential Risk, Cambridge University (Academic). | Participated in all stages. | 0 |
| Gorm Shackelford | Department of Zoology, University of Cambridge (Academic). | Participated in all stages. | 1 |
| Derek Smith | Professor of Infectious Disease Informatics, University of Cambridge (Academic). | Participated in all stages. | 0 |
| Nicola Spence | Department for Environment, Food and Rural Affairs (Defra) (Government). | Participated in all stages. | 40 (the questions were developed with a single focus group). |
| Helene Steiner | OpenCell, London (Private Sector). | Participated in all stages. |  |
| Lalitha S. Sundaram | Centre for the Study of Existential Risk, Cambridge University (Academic). | Participated in all stages. | 5 (specific questions from 3). |
| Sam Weiss Evans | Program on Science, Technology, and Society at Tufts University (Academic). | Participated in all stages. | 0 |
| Silja Voeneky | Department for Public International Law, Comparative Law, and Ethics of Law, Law Faculty, Freiburg University (Academic). | Participated in all stages. | 5 (3 provided questions). |
| John R. Walker | Arms Control and Disarmament Research Unit, Foreign and Commonwealth Office (Government). | Voted, participated in workshop and helped with drafting. | 0 |
| Harry Watkins | Department of Landscape, Arts Tower, University of Sheffield (Academic).  Rootstock (NGO). | Participated in all stages. | 12 (comments from 2). |
| Simon Whitby | Bradford Disarmament Research Centre , University of Bradford (Academic). | Participated in all stages. | 0 |
| James Wood | Department of Veterinary Medicine, University of Cambridge (Academic). | Participated in all stages. | 0 |
| William J. Sutherland | Department of Zoology, University of Cambridge (Academic). | Participated in all stages. | 2 |
| Thomas Meany | OpenCell, London (Private Sector). | Preparation of questions and editing draft. | 0 |
