# Supplementary material for: 80 questions for UK biological security
Source: PLoS One. 2021 Jan 6;16(1):e0241190. doi: 10.1371/journal.pone.0241190 (PMC7787535; doi:10.1371/journal.pone.0241190)
Supplement: S2 Appendix — (DOCX) [file pone.0241190.s002.docx]

# 100 Questions: Round 1 Compiled List

## Bioengineering and Novel Technologies

1. What are the possible biosecurity risks for the UK from widespread use of CRISPR-cas9?
2. Do advances in custom DNA synthesis technologies, and their widespread availability, pose novel biosecurity threats to the UK from human-engineered agents, and how can these be mitigated or prevented?
3. Should DNA synthesis machines be tracked and restricted to licensed companies, non-profits, or government entities in the UK?
4. Do genome editing approaches pose a threat to UK biosecurity?
5. How does the U.K. respond to unregulated bioengineering/gene editing research being carried out in other countries?
6. How do we measure, interpret, report and record off-target events and their effects in CRISPR-Cas9 genome editing?
7. What safety standards would need to be met to demonstrate that therapeutic genome editing of germline cells is safe and effective for use in the population?
8. Even with a “100% safety” guarantee assuring no off-target effects, should germline genome editing be used in the population?
9. How close are we to being able to design fully synthetic microorganisms for a dedicated purpose? (e.g. degrading plastics in the environment).
10. What is the easiest (most cost-effective) way to make a biological weapon in the UK?
11. What are the most relevant emerging innovative technologies to UK biosecurity?
12. Do synthetic organisms pose a plausible threat to the UK?
13. What will be the consequences for the UK of making synthetic human genomes?
14. Can traditional biocontrol agents (such as parasitoids) be safely engineered to be more host specific?
15. Are species-specific herbicides feasible?
16. Is it possible to design a recallable gene-drive system?
17. What pathogens can be “biohacked” in a garage laboratory within the UK?
18. What impact have new and emerging biotechnologies had on the ability of so-called ‘garage biologists’ or ‘DIY biologists’ to create or adapt potentially dangerous biological pathogens?
19. How can we best encourage a culture of safety and security in DIY bioengineering?
20. Can real-time DNA-based wind-carried spore sampling networks be constructed economically?
21. Can affordable DNA-barcode-based hand-held identification machines be built in the UK (for, e.g., customs officers)?
22. Does the UK have sufficient high containment facilities (BSL4) to allow us to undertake research into highly virulent pathogens?
23. De novo synthesis of new pathogens or old pathogens with ‘gain of function’ mutations are now possible with new technologies, and can be done fairly easily by someone with basic molecular biology skills. How can technologies be developed for detecting and containing such new (currently non-existing) pathogens?
24. Synthetic biology approaches to population control (e.g. of insect vectors or pest species) are now being developed. How can the risks posed by and potential benefits of these approaches be assessed rationally?
25. Which particular science and technology developments are most relevant and likely to have impact in the UK in the near future?
26. What adaptations are necessary to existing UK risk analysis processes for synthetic biology products?
27. What modifications are needed in the UK so that GMO deliberate release regulations can meaningfully apply to gene drive organisms?
28. How are current DNA synthesis operations governed? In the UK, globally, and at the intersection (i.e. shipping)?
29. Is there a specific ethic of biotechnology/biosecurity in the UK?
30. Is there a need for a risk assessment procedure (backed by hard or soft law) before starting (and during) biosecurity relevant research?
31. Should the operator that causes a biosecurity threat because of its biotech activities be liable to pay the costs of the response action?
32. What are the risks from gene-drives being used in other areas but introduced or naturally spread elsewhere?
33. If benignly-intended work is being conducted by those only with knowledge and skills relevant to safety in the laboratory (re. biosafety, biosecurity and biorisk management), what about the wider biological security implications of such work?
34. Is it ethically acceptable for research innovation to take place without taking into account wider ethical and social issues (CRISPR-cas9 babies may be an example of ethical and social issues of vital importance)?
35. How will environmental DNA approaches be integrated into surveillance?
36. How do we use smart technologies to monitor human, animal and plant exposure to pathogens in the UK?
37. How do we effectively decontaminate complex (imaging in particular) equipment in order to apply state of the art technology to the study of high consequence pathogens? (This project would have to have close links with instrument manufacturers).
38. How can new biological and computational technologies be leveraged to enable continued trade in living materials such as seeds, plants and livestock, while reducing the risk of pathogen transmission?
39. How can ongoing advances in technology (e.g. biosensors, rapid diagnostic metagenomics etc.) be rapidly and responsively integrated into existing plans for national responses to newly emerging agricultural (plant and animal) pathogens?
40. How can we harness the power of new sequencing technologies to create a genuine ‘One Biosecurity’ surveillance capability?
41. How do we ensure that proportionate action is taken on diagnosis data from high throughput sequencing e.g. are there genetic signals that would indicate the threat posed by a previously unknown species; can the DNA of a species tell us about its potential impact on all hosts?
42. How can we make the emerging technology of DNA storage secure and safe?
43. How can digitalization and/or the use of Artificial Intelligence (AI) systems help to improve biosecurity?
44. Could and should there be an automatic alert system in case of a biosecurity emergency?
45. Could there be an AI driven screening of online papers published with regard to biosecurity relevant information to warn or inform UK government or the public?
46. Could there be an AI driven alert system predicting biosecurity emergencies; if yes, who should get the information?
47. Is the UK public’s understanding of research in bioengineering adequate to underpin democratic decisions on biosecurity?
48. Are UK policy-makers sufficiently equipped with evidence and understanding to make decisions in this area?
49. Are current international import and export regimes - as exemplified by the Australia Group – capable of responding to the complexities of synthetic biology and other new and emerging biotechnologies?
50. Given the feasibility of creating fully synthetic bacteria (e.g. Syn61), and the open-access availability of sequenced and annotated bacterial genomes, how easy would it be for an AI system to design and implement a fully synthetic microorganism for a dedicated purpose?
51. Do synthetically recoded bacteria and similar advances present new challenges for biosecurity and biocontainment? (e.g. https://www.nature.com/articles/s41586-019-1192-5)
52. Is the Biological Weapons Convention (BWC) fit for purpose given new bioengineering technologies? If not, what reforms would be needed to ensure it meets its objective in a world of new bioengineering technologies?
53. Could new technologies aid monitoring and verification under the BWC? If so, what are these technologies and barriers to deployment under the BWC?
54. What are the major pathways by which a biologically engineered threat could enter the UK?
55. What are the base rates of an accidental or intentional release of bio-threats from UK labs?
56. Do bioengineered threats require distinctly different eradication measures in comparison to previously used techniques?
57. Could de-extinction technologies (and resurrected species) be a biosecurity risk for the UK in the future?
58. What principles and policies (if any) should the UK adopt in relation to de-extinction technologies?
59. To what extent has the convergence of recent advances in life sciences with cyber-security threats led to an increase in cyberbiosecurity vulnerabilities and threats?
60. How best can we anticipate cyberbiosecurity threats and mitigate their effects?
61. What analysis is required: a) fully to scope cyberbiosecurity and to identify a comprehensive strategy to tackle it? to b) to examine causes, effects and define possible remedies?
62. How best can experts engage key government agencies to raised awareness of and initiate changes to relevant policies and programmes to include relevant cyberbiosecurity measures.
63. What communities have the responsibility to ensure the responsible development of bioengineering technologies in the UK?
64. What regulation should there be around individuals biohacking their own bodies?

## Dual-Use

1. How can we measure the success/failure of biosecurity interventions in the UK and adaptively use this to improve our responses?
2. What are the most effective/efficient methods for raising awareness and changing behaviours in favour of better biosecurity practices?
3. How can we make cost-effective decisions on the proportions of an available budget to spend on prevention, response or on-going management?
4. How do we make the opinions of academics more relevant to policy?
5. How effective, transparent and timely are the mechanisms in the UK for monitoring and controlling research with potential for dual use?
6. Are our regulations regarding the dual use of research sufficiently robust to prevent the inappropriate use of research?
7. What measures can be taken to ensure that the private sector does not explore dangerous dual-use technologies without societal approval?
8. Does biotechnology need to be dual-use to be a dominant technology of the twenty-first century in the UK?
9. Are existing measures within the UK adequate to prevent the exploitation and use of chemical, biological and toxin weapons in future wars?
10. What are the driving forces likely to lead to the widespread hostile use of biological and toxin weapons in coming decades (e.g., advances in science and technology, the changing nature of warfare and the decline in the use of multilateral agreements), and how might these forces best be countered?
11. How can the dual use awareness and education of biotechnologists be improved over a reasonable timescale, so that their expertise is available to help protect their benignly-intended work from such misuse, without the development of an International Biological Security Education Network similar to the IAEA-funded International Nuclear Security Education Network?
12. What type of governance systems, and assessment procedures, needs to be in place to address dual use research concerns and ensure a balance between benefits and risks?
13. What regulatory and/or guidance frameworks and mechanisms (including legislation, regulations, guidelines, standards, technical measures, funder reviews and oversight, codes of conduct, education, and self-governance) are available (or could be created) to draw on when developing dual-use policy and best practice?
14. Can we draw lessons from other fields of risks/dual use governance for the field of biosecurity? If so, what lessons?
15. What is the feasibility for misuse of specific technologies within the UK? What aspects should be focused on (e.g. regulating the products or the underlying technology)?
16. What range of activities need to be considered when developing dual use policies in the UK?
17. Is the application of a dual-use technology in the UK dependent on the simultaneous development of other technologies?
18. What are the considerations for intangible technology, such as tacit knowledge and the accessibility of the knowledge base for potential misuse?
19. Do policy approaches to dual use in the UK need to be specific for individual technologies, or could they be wider-based to allow assessment of risks for all relevant emerging technologies?
20. Who should be involved in predicting the implications of dual-use technologies in the UK? How do we reflect different interests and capture a broad range of voices to clarify perceived risks and weigh the benefits and risks?
21. Wo what extent can dual-use policy across different sectors be harmonised?
22. Would it be useful to develop guiding principles for balancing the benefits of scientific research with the responsibility to prohibit and prevent the development and acquisition of biological weapons? What aspects would such principles cover?
23. When should decisions on governance be made and at what stage of technology readiness (from initial research through to product) should they be applied?
24. How can we ensure that proportionate and adaptive governance measures are considered in dual-use biosecurity policy in the UK?
25. Are there existing measures that adequately cover, or could be extended to cover, relevant technological dual-use advances in biosecurity in the UK?
26. What is the level of awareness in industry and in industrial policy of dual use research of concern issues?
27. Does laboratory research on antibiotic-resistant and anti-microbial bacteria raise concerns similar to those under discussion regarding gain-of-function virus research, and are researchers aware of these dual use concerns?
28. What are key markers of “weaponisation” of biological agents and are these monitorable?
29. Who decides what is dual use in UK biosecurity?
30. How do we ensure that any new regulations/policies/laws brought about to mitigate biosecurity risks(s) have minimal negative impact on academic research?
31. How do we ensure that solutions to dual-use biosecurity concerns in the UK are not worse than the problems they aim to solve?
32. Are there positive or beneficial consequences to the discovery of pests or pathogens?
33. Are the risks of gain of function research on influenza virus outweighed by the benefits of such research?
34. Which scientific/technological directions for rapid identification of infectious biological agents present most promise for identification of emerging disease threats? (E.g. see https://www.openphilanthropy.org/blog/explaining-our-bet-sherlock-biosciences-innovations-viral-diagnostics)
35. Are there any effective methods from other fields for examining the potential benefits and risks of research to inform whether research should be funded/published?
36. Should the UK require biological engineering laboratories to acquire insurance for potentially catastrophic outcomes?
37. What conditions would necessitate establishing Biosafety Level 5 (BSL 5) facilities in the UK
38. What would be the most appropriate and effective safety conditions and practices for a BSL 5 facility in the UK?
39. How should the UK deploy the precautionary principle in relation to UK biosecurity? In particular, what are the thresholds for plausibility and irreversible damage?
40. What are the benefits and risks associated with using AI systems in bioengineering in the UK?
41. By what metrics should we estimate and compare the potentially costs and benefits and risks of bioengineering advances (money, quality-adjusted life years, happiness and wellbeing etc.)?

## Pandemics and Epidemiology

1. What amino acid substitutions are necessary to make avian influenza viruses transmissible among humans and cause an influenza pandemic? (This needs to be done for at least the H2 H5 H7 H9 subtypes, and for multiple genetic lineages within each subtype; the question is already answered in part for two lineages of H5 viruses, but for proper risk assessment needs to be answered more comprehensively.)
2. What are the within-host evolutionary pathways for avian influenza viruses to evolve within a mammalian host to adapt to transmission within humans?
3. In an avian influenza infection in human (done using the ferret model), how much virus is excreted, and are partially adapted-to-human mutants preferentially excreted (for example those with some affinity for the upper respiratory tract)?
4. Based on the phenotypic changes need for an avian influenza virus to adapt into a pandemic influenza virus in humans which of these changes exist in currently circulating avian influenza viruses worldwide?  (There should be substantial phenotyping involved in answering this question until the genetic bases of the necessary phenotypic changes are comprehensively catalogued).
5. What is the risk of an influenza pandemic?
6. How pathogenic to humans are different, plausible influenza threats? (An estimate of pathogenicity is key in subsequent planning based on a risk assessment).
7. How could a process of understanding phenotypic changes, amino acid substitutions and evolutionary pathways that allow for human transmissibility to different biosecurity threats in the UK?
8. How should we apportion funding and other resources to most effectively mitigate the risk of known (re)emerging infectious diseases given existing risk assessments?
9. What approaches are possible to protect against currently unknown emerging infectious diseases, and how much funding and resources should we apply to these diseases compared to known (re)emerging infectious diseases?
10. How much can our understanding of niche availability and other barriers that invasive species face in the macro-world inform risk assessment in the pathogen world of the UK?
11. What under-researched but novel broadly applicable approaches could help us prepare for pandemics in human diseases we are not specifically prepared? (E.g. could sneeze or cough suppression drugs help to substantially reduce transmission of respiratory diseases)?
12. How can modelling better simulate the potential impacts of deliberate release events and of possible response interventions in the UK, given that population behaviour in these events will differ from naturally occurring outbreaks?
13. Can retrospective modelling techniques be developed which allow investigators to rapidly identify possible transmission routes of deliberate release events in the UK (‘reverse epidemiology’)?
14. How can syndromic surveillance thresholds in the UK be improved to more effectively separate signal from noise for unusual events and reduce time to alert without overburdening the system?
15. Which new methods or technologies could be employed with the information currently collected in the UK’s syndromic surveillance system to get a clearer, faster alert of an unusual event or number of cases?
16. What tools could most effectively improve the detection of ‘slow-burn’ epidemics (eg. HIV, vCJD, cancer-causing pathogens such as HPV)?
17. How can collaborative work between agencies (eg. PHE, DEFRA) be improved and better address ‘One Health’ risks?
18. What is the risk that the UK would be unable to increase the human resources required in health, veterinary and vector control services at the speed necessary to treat patients and control a serious outbreak? Which factors most influence this capacity and what mechanisms could best mitigate them?
19. How can the UK deliver antivirals to pandemic flu patients rapidly enough for them to be effective (within 24-48 hours of symptom onset)?
20. Can the UK develop a non-pandemic-specific influenza vaccine that can be used to prevent or mitigate a pandemic of influenza? (Note, a pandemic-specific vaccine is likely to be available too late to have a significant impact on the first wave of a flu pandemic).
21. What are the most effective delivery mechanisms for universal flu vaccines, and what governance should there be of emergency vaccines programmes?
22. Which correlates of protection can be used to assess protection by new universal flu vaccines in the UK?
23. Can we develop a high throughput, non-specific, non-invasive rapid mass screening test able to detect infection before the infection is transmissible and/or before symptoms manifest, to replace checkpoint/border fever screening in outbreaks?
24. There is currently no legal basis for initiating quarantine as an intervention in the UK. If it were required, how could it be effectively implemented? What population reaction could be expected in the face of home quarantine and which actions would most effectively achieve compliance?
25. Could a set of generic protocols and associated biobanks be effective in improving the speed of confirming whether newly-identified zoonotic diseases have potential to be clinical/public health threats?
26. What proportion of health facilities currently practice the level of Infection Prevention Control necessary to be sure that an infectious outbreak would not be amplified in the facility, and beyond?
27. In the event of the deliberate release of an airborne pathogen, for how long would different pathogens contaminate the environment and what decontamination methods will be most effective?
28. Is the current method of assessing and allocating hazard levels to known and novel pathogens producing policy and guidelines that are fit for purpose in guiding IPC in practice in UK healthcare facilities and laboratories? How could additional concepts such as High Consequence Infectious Diseases support more efficient and standardised IPC in practice.
29. Is the UK strategic, technical and operational response to pandemic threats fit for purpose? Is it consistent with what is known about the biggest human threats, namely directly-transmitted viral infections such as influenza or a coronavirus?
30. Has the UK strategic, technical and operational response been tested/gamed/simulated operationally?
31. What are the consequences of failing to contain the rise of antibiotic resistant infections (bacteria, and some viruses like HIV), and what are the best methods of containment? (This question was not honestly answered by the O’Neill report (or the World Bank), both of which confused science with advocacy).
32. How will biosecurity risk management change as a consequence of Brexit and what measures must be taken to compensate and at what cost?
33. What is the potential for state-of-art genomics to characterize and more effectively prevent/contain infectious disease outbreaks? Is genomics an effective addition to the practitioner’s toolbox, or is it an instrument of more fundamental research?
34. Is biosecurity risk correctly evaluated, and how best can risks be communicated to public and policy-makers? [This is about the computation and communication of statistics, real and fake, truthful and dishonest. Major points of contention here]
35. The foot-and-mouth virus has never been used in bioterrorism, despite its seeming availability; what is the reason for this?
36. How can we best harness the expertise in manpower within academic institutions for the control of epidemics here in the UK and abroad?
37. Should we be establishing a reserve of lab scientists that could deploy as part of the UK rapid response team to provide laboratory support during epidemics?
38. Is the UK doing everything it can to support epidemic response at home and abroad?
39. Are the screening procedures at our borders sufficient to prevent/limit the importation of exotic human and/or animal pathogens?
40. Can we better harness rapid tests (or new technologies) to improve border security - specifically in relation to animal pathogens?
41. What factors limit onwards transmission of pathogens from global transport hubs?
42. How do we judge the degree of response vs pathogenicity?
43. What impact has the emergence of networks on the dark web had on the risk and proliferation of potential biological weapon agents?
44. To what extent can social media platforms be integrated into our early detection/warning systems?
45. Are there technologies on the horizon which may lower the barrier of effective delivery of biological weapon agents and how do we mitigate against their misuse?
46. Can 'amateur' biologists really reproduce experiments based solely on video footage?
47. Would an influenza outbreak with similar characteristics to the 1918 influenza virus (in terms of infectivity, lethality) have a greater or lesser global spread and mortality if it occurred in 2020?
48. Which animal reservoirs of influenza virus variants should currently be of most concern from a human health perspective in the UK (e.g. seal, avian, swine)?
49. What are the key plausible steps that would have allowed a faster identification of, and more timely and effective response to, the 2014 Ebola outbreak?
50. Should there be increased international funding for pandemic surveillance and preparedness? What funding models would be most appropriate?
51. Historically, what have been the most effective interventions for preventing the spread of invasive pathogens or reducing their impacts in the UK?
52. What are the most cost-effective interventions for stalling or reducing anti-microbial resistance in the UK? Why have these not been adopted?
53. What are the mortality base rates (fatalities per person per hour of exposure) for pathogens within the UK?
54. How can we (academia) best support the government through advocacy to prioritise pandemic preparedness?
55. How can we (academia) best identify gaps in the UK’s pandemic preparedness?
56. What have been the key faults and pathways that have led to disease spread and establishment in the UK?

## Environment, Plant Disease and Zoonoses

1. Are environmental (i.e. biodiversity and ecosystem) impacts of harmful organisms given sufficient weight (in comparison to social and economic impacts when comparing biosecurity threats?
2. Is there a biosecurity risk from importing plants rooted in soil from China (and elsewhere) and if so what is it?
3. What research infrastructures could be put in place to allow international teams to work together to respond to newly emerging crop diseases?
4. What are the best tools to identify the source of wood (tree species and their origin) or other plant material in a mixed consignment?
5. Which tree diseases are most likely to become more widespread?
6. What novel options are there for dealing with tree diseases?
7. Can disease-resistant trees be produced? If so, what is a realistic time scale for roll out and at what likely magnitude?
8. Are there realistic and sensible policy responses to the widespread distribution of plants via internet sales?
9. When, if ever, is salvage logging a sensible response to tree diseases?
10. Is it possible to assign liability for the supply of infected plants or materials, and what would the impact on procurement and supply chains be if it is?
11. Is it possible to engineer or select biosecure plants?
12. What are the social/cultural impacts of plant and animal pathogen outbreaks?
13. What are the cumulative or multi-trophic risks associated with pests and pathogens that affect plant species?
14. What are the true associated risks of moving shellfish from one waterbody to another?
15. Will we be able to maintain ‘bonamia’^[[1]](#footnote-1)^ free waterbodies in the UK?
16. Can we build resilience to bonamia? [ZSL]
17. What are the emerging zoonotics of concern to the UK?
18. How do producer and operation demographics and risk attitudes and perceptions affect biosecurity response plan adoption?
19. How can producer and operation demographics be altered to minimize the expected effect of negative animal health and supply chain consequences of animal disease outbreaks?
20. What will be the effects of climate change and Brexit on trade patterns and pathways?
21. What is needed to develop and deploy a global trade model relevant to the UK that can incorporate climate change effects?
22. How should we best allocate scarce inspection resources to maximise the probability of detecting quarantine risk materials, while also learning about changes in prevalence and composition on pathways and providing a degree of deterrence?
23. What disease risks are associated with the changing ecology of (insect) disease vectors?
24. What determines the host range of oomycetes (Phytophthora etc.)?
25. How will climate change influence migration and hibernation of animal pathogen reservoirs?
26. How will urban heat sinks/architecture/behaviour influence pathogen transmission?
27. How is baseline surveillance of the UK flora and fauna used to predict risk?
28. There has been a recent court case in Canada where someone was fined for bringing undeclared samples of serious animal pathogens into the country in their checked baggage. Do UK border controls have mechanisms that would have picked up such activity?
29. Do honey bee pathogens affect other pollinator species, if so, to what extent?
30. Can diagnostic tests and models forecast disease development in honey bees?
31. How do you get farmers and animal keepers to follow best biosecurity advice during periods when epidemic risk is not enhanced?
32. Do carrot or stick approaches work best as a means to reduce risky trading practices for diseases for which there are statutory controls with compensation? (Of relevance for bovine tuberculosis controls in England, Ireland and Wales i.e. is reducing compensation for risky trading practices more effective than providing certification for those farmers behaving the best?).
33. How do you reach relatively inaccessible elements of the animal keeping population with biosecurity advice? (Of particular current relevance for small scale pig keepers in relation to African Swine Fever risk in UK).
34. What are the economic and cultural drivers of pig keeping practices in the small scale/ marginal commercial, hobby and pet sectors, especially in relation to feeding and biosecurity?
35. How can the registration of novel tests for animal diseases be accelerated?
36. How much protection does being an island provide to UK?
37. How does the current framework deal with issues of ecocide in the UK?
38. What is happening with UK soil integrity, is this something that is being monitored?
39. What changes to agricultural policies can be made to incentivize the preservation and restoration of biodiversity necessary to prevent mass extinctions/deforestation while maximizing progress towards the 71% increase in agricultural production required by 2050 to achieve food security?
40. How can assessments of biodiversity be used to assign monetary value to ecosystem services in the UK to objectively inform land-use policies?
41. Can improved regulation of the trade and supply of agricultural products be used to prevent or mitigate threats to national security posed by spikes in food prices following localised crop failures due to environmental factors such as droughts, floods and diseases?
42. Which farming systems in the UK are most vulnerable to biosecurity risks?
43. Which food supply chains in the UK are most vulnerable to biosecurity risks?
44. Which species or habitats do we most need to conserve in order to maintain ecosystem functioning?
45. How accurately can we predict timescales and extent of landscape change as a result of biosecurity outbreaks?
46. Is it possible to identify high or low risk landscapes?
47. How different are the micro climates that pests experience to the climates recorded at Metereological stations?
48. What is the role of ‘managing landscapes’ as part of a biosecurity strategy?
49. Can overwintering potential of pathogens be inferred from simple temperature-related relationships?
50. Can we improve predictions on wildlife diseases?
51. What lessons can be learnt from human, animal and plant health biosecurity that are applicable to wildlife diseases?
52. How can we improve the effectiveness and cost-efficiency of wildlife disease surveillance?
53. Can stable isotope analysis be used to track provenance of living plant material?
54. Could block chain or smart contracts be used to trace ownership or provenance of plant materials?
55. How can plant species with recalcitrant seeds be safely biobanked?
56. Can appropriate surrogates be identified should a species be lost from an ecosystem?
57. Can the grey literature (such as Environmental Impact Assessments) be harnessed to provide early warning for pathogen pathways or identify high risk landscapes?
58. Which disease-carrying vectors are most likely to spread to environments with significance to UK and European biosecurity as a result of climate change over the coming 20 years?
59. What new or enhanced biological threats will the UK face in a world of catastrophic climate change (4°C of warming by 2100)?
60. What new or enhanced biological threats will the UK face in a world in which temperatures have risen by 2°C (by 2100)?
61. How does biodiversity loss, in particular the decline in pollinators, affect the spread and potency of animal, plant and environment pathogens?
62. What infrastructure and economic systems are most vulnerable to biosecurity threats?
63. How would different, plausible climatic tipping points (permafrost thawing, increased oceanic bacterial respiration, Amazon and Boreal forest dieback) effect UK biosecurity given the increased rate of temperature change?
64. What would be the impact of stratospheric aerosol injection (a geoengineering technique to lower global temperatures) on UK biosecurity?
65. How do we improve prevention, detection and control of disease threats to species conservation in the UK?

## Invasive Species

1. Can we develop scenarios to indicate how many new invasive non-native species, pests and pathogens may establish in the UK by 2050 or 2100 (considering trends in their major drivers such as climate, land use, trade, transport, governance etc.) and what their environmental and socio-economic impact will be? What management approaches would best minimise these impacts?
2. Have the different UK biosecurity regimes (e.g. animal health, plant health, fish health, bee health, invasive non-native species) been successful? If not, what have been the barriers to success?
3. How can we compare the threat from species that cause delayed chronic impacts (i.e. impact does not occur until tens of years after establishment, but is then persistent – often the case with invasive species) to those that have more acute threats (often the case with pests and pathogens of plant and animal health)?
4. How should we sample and inspect introduction pathways of invasive non-native species to gain a better understanding of a) the risk they pose and b) how to reduce their risk?
5. How can we predict which non-native species will become invasive (i.e. cause impacts)?
6. What future changes to trade, transport and travel pose the greatest threats to the UK in potentially introducing new invasive non-native species, pests and pathogens?
7. Can the invasive non-native species (INNS) zebra mussel be used as cultch without biosecurity risk? If so, what is the process that that should be undertaken? (It is currently a waste product from the water industry but has associated risks from itself but also because it hosts the killer shrimp which effects fish populations.)
8. Can longitudinal random sampling of ticks and other vectors arriving into the UK on domestic animals be done to track vector-imported pathogens that could take hold in UK domestic species, and imported species with the potential to establish presence in UK environments?
9. What is the current distribution of Aedes albopictus in the UK and the rate of arrival and survival?
10. How can monitoring and mapping of other new invasive vectors (midges, ticks) affecting livestock and/or humans be improved?
11. Which of the biosecurity risks facing the UK (e.g., invasive species, agricultural pests and disease vectors) might be usefully addressed using modern methods of population genetic control, including gene drives?
12. What are the predictive attributes of invasive species with regards to invasion stages i.e. transport, establishment, spread and impact?
13. What are the current propagule pressure patterns in UK?
14. What are the current colonisation pressure patterns in UK?
15. What are emerging UK pathways and vectors for invasive species?
16. What are the most effective eradication and control measures for primary and secondary spread in UK?
17. What patterns of connectivity present and future will facilitate/hinder spread?
18. What is the relationship between invisibility and habitat degeneration/habitat improvement?
19. What is the relationship between biodiversity and invasibility in UK?
20. Will rewilding enhance or reduce invasions in the YK?
21. Is an invasion meltdown (large-scale economic and ecosystem services losses) inevitable in UK?
22. Are eDNA technologies likely to keep pace with demand for this method?
23. How can social media best be used in detecting and monitoring invasive species?
24. What risk do invasive species pose in terms of pathogen introduction and transmission?
25. What will be the impact of Brexit (or other changes in trading patterns) on the risk of introduction of pathogens/invasive species to the UK?
26. Does latitude matter in predicting the impact of a pathogen/pest incursion?
27. Does Northern Ireland’s position on the extreme western edge of Europe and the unique marine ecosystems that prevail here create distinct vulnerabilities to invasive species?
28. Which species and their potential vectors which Government should examine as part of an invasive species strategy in Northern Ireland?
29. Is there a limit to the number of invasive species that can establish in particular UK habitats? (If so are we approaching that limit or have we only just begun?).
30. How effective are different means of predicting which species are likely to invade a region?
31. What has been the success of horizon scanning activities for predicting and managing invasion?
32. What has been the success of testing the ecological impact of species intentionally introduced, such as for biological control?
33. Do educational programmes result in any change in behaviour of those who might transmit invasives?
34. How important are pot-grown plants as a source of invasive species?
35. Which species are likely to as arrive as more shipping uses the arctic route to the Pacific?
36. Have the serious measures for dealing with biosecurity at entry to Australia and New Zealand been effective?
37. How can early warning systems to deal with arriving invasives be made more effective?
38. Can forestry systems be made more resilient to future invasive species?
39. What determines the optimal allocation of expenditure in (i) preventing entry, (ii) early identification and eradication, (iii) controlling and (iv) mitigating impacts of invasives?
40. Which management practices are most effective and/or cost-effective for controlling invasive species?
41. What evidence is there that border controls are effective at preventing biological invasions?
42. What evidence is there, if any, that border controls are more effective than controlling/eradicating invasive species after they become established?
43. Which management practices for invasive species would have positive or negative impacts on other policy areas? Which impacts and how large?
44. Which management practices for invasive species are most acceptable to different groups of stakeholders? (E.g., culling, quarantine, chemical control, biological control?).
45. How can predictions of invasive species impacts be regularly validated/updated?
46. How does invasive species control affect other environmental or societal outcomes?
47. Is citizen science a reliable method of monitoring species invasions?
48. What would be the differences between an intervention and non-intervention (of invasive species) scenarios?
49. How can policy and management steer the situation towards a particular future of invasive species?
50. How much invasion can we avoid through management?
51. What are the impacts of invasive species on Nature’s Contribution to People?
52. Do current risk assessments cover the impacts of invasive species on ecosystem services and Nature’s Contribution to People?
53. Is there any evidence of invasive species declining anywhere as a result of climate change allowing them to expand towards the poles?
54. What climate migrants can we expect in the upcoming decades?
55. What are the consequences for biological invasions in the UK under 1.5ºC of global warming?
56. What are the consequences for biological invasions in the UK under 2ºC f global warming?
57. Could climate change mitigation policies (e.g. water transfers, changes in crops, biofuels, re-forestation) promote invasive species?
58. Could climate change adaptation policies (e.g. water transfers, changes in crops, biofuels, re-forestation) promote invasive species?
59. How can we make scenarios of invasion relevant for stakeholders?
60. Is there any empirical evidence of the role of protected areas as filters against invasion?
61. Do species with high vs. low impacts have particular traits? Can we use them to horizon scan prospective invaders?
62. Can the new artic transport routes promote biological invasions?
63. Are military activities a potential vector of invasive species? If so, are they disregarded?
64. How can we better predict the potential for species to establish and spread in novel environments?
65. What is the true impact (economic, societal and species) of current invasive wildlife diseases in the UK?
66. What are the most effective ways to combat non-native wildlife diseases once they have been introduced into a country?
67. How do we prevent the introduction of wildlife diseases to novel geographic regions or species in the UK?
68. How do we apply the same level of sanitary protection to imported wildlife as applies to our livestock and fish industries and which specific pathogens (identified as threats to UK biodiversity) should be tested for?
69. How well is the UK and its overseas territories managing the impact of invasive species and controlling the risks of further invasion?
70. Which invasive species that are already in the UK pose the greatest harm to a) human health; b) animal health; c) plant health; and d) biodiversity?
71. What actions should the UK take to mitigate the risk, or adapt to, climate migrations of invasive species?
72. Where should the four nations prioritise resources to tackle invasive species?
73. How may future changes in trade patterns affect the UK’s ability to predict and manage invasive species?
74. How can the risk of trade and future trading relationships bringing non-native invasive species to the UK be mitigated?
75. How effective have the European Union’s Invasive Alien Species Regulations been at addressing and tackling invasive species?
76. In the event of EU exit, how should the UK establish its replacement for the European Commission’s scientific forum to update the species list of concern?
77. Does Brexit offer any benefits for our approach to invasive species management?
78. How good are we (the UK) at managing pathways for invasive species?
79. How effective is UK border control in managing entry of invaders?
80. How will we deal with the increasing important of ecommerce in managing the introduction of invasive species?
81. How can we better foster the involvement of the public in the monitoring of invasive species?
82. How frequently should we conduct horizon scanning events for invasive species in the UK?
83. What are empirically the most effective interventions at either preventing invasive species introduction or reducing their impacts?
84. What are the major pathways by which invasive species or pathogens could enter the UK?
85. What are the base rates of introduction of invasive species into the UK; introduction of invasive pathogens into the UK?
86. What have been the most cost-effective eradication measures within the UK for invasive species and pathogens?
87. What have been the most cost-effective eradication measures internationally for invasive species and pathogens that are relevant to UK biosecurity?
88. What actions and guidelines would minimise the chance of invasive species release and spread during research activities?
89. Should research activities on invasives be potentially stopped when there is a high chance of accidentally releasing or spreading invasives?

## International and Domestic Law and Policy

1. How can we assemble international data on management efficacy to best inform decision making in the UK?
2. How can we encourage more systematic research into effective and/or novel management measures, including documenting the findings of practitioners in the field?
3. Is there adequate sharing of information, resources, capacity across biosecurity regimes?
4. How should we assemble international data on new and emerging risks to make horizon scanning a more quantitative exercise?
5. How do we define best practice for biosecurity to match the varying risks and resources of the world’s nations?
6. When using recycled shell (e.g. shell as a waste product from food industry) what is the best process to ensure there are no biosecurity risks from its redeposition in the sea? (The currently practice is to leave exposed for 6 -12 months but it has not been test).
7. What may be done practically within the context of the Chemical Weapons Convention (CWC) in the next five years to strengthen the prohibition against the hostile use of lethal chemical agents?
8. Do other more diverse means outside of the Biological and Chemical Weapons Conventions now have to be taken in order to prevent the hostile application of biotechnologies?
9. What new means might be developed to make the operations of the Biological and Toxin Weapons Convention more effective (e.g., majority voting, more administrative staff, stable funding) and how can the impact of these changes be assessed?
10. Given the inability to agree on the question of verification measures for the Biological and Toxin Weapons Convention how far can development of agreements on other aspects of the Convention such as assistance (Article VII) and cooperation (Article X) prevent the erosion of confidence in the Convention and how might that improvement in confidence be measured?
11. Is the future role of the Biological and Toxin Weapons Convention most likely to be that of a norm setting agreement and thus policy developments and implementation are more likely to be developed by other mechanisms outside of the direct remit of the Convention?
12. Are risk assessments on other aspects, such as safety and ethics, of relevance to biosecurity or to implementation of the BTWC?
13. What international partnerships and platforms could be put in place to improve the detection and management of plant pathogens before they reach the UK/EU?
14. In order to accelerate the discovery of bioactives and genetic diversity to improve human health and agricultural productivity, what incentives could be put in place to encourage the sharing of information relevant to the conservation and sustainable use of biological diversity (as mandated by Article 17.1 of the Convention on Biological Diversity) while still preserving commitments to benefit sharing?
15. When the UK uses official development assistance to e.g. to support new laboratory or clinical facilities, what provision is made for ongoing maintenance to sustain the biosecurity of such sites?
16. Are different understandings of the term biosecurity problematic for coordinated action across the UK government and for effective engagement with expert communities?
17. When do we apply the precautionary principle with regard to biosecurity issues?
18. Should the UK require its laboratories and operators to maintain adequate insurance or other financial security, such as the guarantee of a bank or similar financial institution, to cover liability?
19. If World Health Organisation (WHO) expectations for good laboratory practice are met, is this training sufficient for insurance and legal purposes?
20. What about wider considerations enshrined in politically- and legally-binding, and internationally mandated regimes, that go beyond the laboratory door such as the BWC, the CWC, UNSCR1540, the Australia Group, and IHR 2007?
21. Do we need a central entity that collects information about biosecurity relevant laboratories?
22. If there shall be liability; shall this be a case of strict liability? If yes: what are the limits of the strict liability?
23. When is expert opinion a reliable source of evidence for biosecurity policy?
24. How can expert opinion and scientific evidence be used systematically to inform biosecurity policy?
25. How can the impacts of biosecurity policies be monitored?
26. How can the trade-offs associated with biosecurity measures be assessed? (E.g., herbicide use vs water quality; bioengineering vs the precautionary principle).
27. What would the impact on biosecurity risk and the economy be of banning the import of soil to Great Britain?
28. Are self-certification schemes (such as Plant Passports) sufficiently robust to ensure the supply of biosecure materials?
29. Is a risk-based approach to policing the biosecurity continuum sufficiently robust given the quantity of goods that pass through British ports and Freedom of Movement within the Schengen Area?
30. How can existing professional roles be adapted to support the ‘Detect’ and ‘Respond’ pillars of the UK Biological Security Strategy?
31. Is laboratory work with dangerous pathogens properly insured?
32. If benignly intended research is misused, what is the insurance situation?
33. If benignly intended research is misused, what is the legal situation?
34. Is it ok for research innovation to take place without taking into account wider legal considerations such as obligations enshrined in national legislation including the BW and CW Acts (in the UK)?
35. What mechanisms are currently in place to support the cross-government coordination necessary for implementation of the UK Biological Security Strategy?
36. What is the most effective, practical control innovation at each of pre-border / border/ post border that could be applied to reduce multiple risks to protect national security, human, animal and plant health?
37. What is the remit of government and non-government UK agencies for biosecurity funding?
38. What are current governmental support structures for institutional biosafety officers (especially post-Brexit)? How might these be improved?
39. How should we regulate (or at least govern) low probability/high risk scenarios that are relevant in the field of biosecurity in the UK?
40. How do we define the field of biosecurity that is relevant for governance?
41. How generalizable are management practices in biosecurity (e.g., across animals, plants, pathogens, bioterrorism)?
42. Are there financial instruments that could be used to offset biosecurity risks (e.g. biosecurity equivalents to carbon markets)?
43. Given the complexity of the stakeholders involved, how much can private-public partnership (e.g. deeds, insurances, assurance schemes) increase responsibility and cost-sharing in biosecurity?
44. Should we think about new ways to incentivize private actors (companies) to limit biosecurity risks? For instance, by obliging them to pay a certain amount of money (depending how risk relevant the business is) to a public fund - if a damage occurs the compensation can be paid by the fund; if there is no damage after 5 or 10 years, parts of the money can be paid back to the company? (Similar to the equity of banks/financial institutions).
45. With large-scale industrial investments in industry being generally limited to established, profitable, biotechnology platforms, what could be done to accelerate investment and development of novel, agile, low-cost systems that could be used for distributed and responsive manufacturing of therapies to combat disease outbreaks?
46. With the aim of increasing agricultural productivity to achieve food and nutritional security, what changes to the regulatory and funding landscapes could be made to improve the rate at which successes in fundamental plant science and biotechnology made in academic institutions are translated into products for agriculture?
47. Do we need a horizon scan of horizon scans?
48. Is what lurks in Bill Sutherland’s beard a biohazard?
49. How general are existing approaches to biosecurity? i.e. are approaches relevant to invasive non-native species applicable to bioengineering? What lessons can be learnt across areas of biosecurity?
50. How temporally dynamic are the various threats? How spatially dynamic are the various threats?
51. What level of coordination operates across the various areas of biosecurity? Are there opportunities for interlinkages?
52. How can we deal with the unknown unknowns in the area of biosecurity?
53. How effective are current methods of ‘community engagement’ in the UK in laying the foundation for the community collaboration and response that will be essential to epidemic control?
54. What can we learn from the failures of community engagement in other countries?
55. Are there untried measures that can be employed to find out how people in the UK would really respond in the face of epidemic control measures?
56. How important is it to ensure effective communication to biosecurity? Should approaches be tailored to different audiences? At different scales?
57. Recognising biosecurity is a shared responsibility, what are the most effective tools to effect desirable behavioural changes amongst the general public?
58. What steps are required to ensure that appropriate education and awareness-raising measures are in place to promote a culture of responsibility in the life sciences?
59. How can communications re biosecurity best be evaluated for effectiveness?
60. How we can modify peoples’ perceptions and behaviours to enhance public support of socially ‘unpopular’ biosecurity measures? (e.g. cutting trees in buffer zones)?
61. How does public perception of which diseases pose most risk in the UK compare with the diseases scientifically likely to have most impact? What can be done to bring perceptions into line?
62. What are the most effective methods to communicate risk of a disease in the UK where name recognition is far greater for diseases unlikely to occur (Cholera, Ebola) than threats most likely to occur (Flu, campylobacter etc.)?
63. What are the most effective methods of anticipating and counteracting the potential for panic caused by media and social media activities in an outbreak?
64. How can we best mobilise citizen scientists to collect reliable and statistically powerful observation data?
65. What is the level of awareness of biosecurity in various sectors in UK, in comparison to other world leaders in the area e.g. New Zealand?
66. What is the willingness to pay for enhanced biosecurity across UK sectors?
67. How do legislation and codes of conduct actually influence individual and group behaviour with regard to biosecurity?
68. Who can I go to anonymously if I have concerns? (Aka why isn’t the U.K. phone line not working?).
69. What happens to any risks I report that do not fit into the current understanding of threats/risk?
70. Is there any activity related to Global Challenges Research Fund life sciences projects that incorporates biosecurity capacity building alongside the scientific capacity building?
71. How are DIY-bio communities engaged with, in the UK?
72. How can we implement biosecurity topics in the curricula of natural scientists? (Students, doctoral students, post docs, and even professors).
73. What can be done to overcome fatalism as a key reason for not taking up biosecurity advice?
74. How can citizen science most cost-effectively be used for biosecurity?
75. Are those involved in biological and chemical research innovation properly trained?
76. What happens to scientific findings that might pose a security risk but are not published (i.e. because they have a negative result)?
77. How do we manage “information hazards” against the imperative to publish headline-grabbing science and the drive towards openness? How can the costs and benefits of open-access research and information hazards be assessed?
78. What happens when an outside company funds research in a U.K. university, and gains knowledge in a biosecurity issue, do they have an obligation to publish?
79. How much do we know about when peer review actually works to ensure that a potentially dangerous piece of research has been turned down?
80. How can we manage biosecurity risks without limiting research in a disproportional way?
81. Is there a need for a risk assessment procedure (backed by hard or soft law) before starting (and during) biosecurity relevant research?
82. How transferrable are research methods in different areas of biosecurity (e.g., control of invasive species vs pathogens vs GMOs)?
83. How could a list of biosecurity priorities be generated? Locally? Nationally?
84. What methods can be used or devised to support the use of evidence in biosecurity policy?
85. How can we work to ensure that a culture of responsibility is baked into life scientists at an early stage?
86. To what extent have government and private industry enacted policies and practices to ensure that biosecurity within their facilities encompasses robust cybersecurity measures?
87. How can we be better prepared to foresee which scientific endeavours may open pathways that will require a new form of governance?
88. How can we better promulgate awareness of the UK national hotline for reporting biosecurity concerns?
89. How can we better engage with industry to ensure that biosecurity measures are implemented?
90. How can we better implement common biosecurity standards and best practices across UK biotechnology facilities?
91. Is there a need for the development of a common ‘seal of approval’ for biological facilities that demonstrate robust and comprehensive biorisk reduction measures, policies and strategies?
92. How important is understanding biosafety and biosecurity for a career in the private sector?
93. Who should pay for biosecurity to protect biodiversity in the UK?
94. What is the cost/risk of bioterrorism in an increasingly post-truth world?
95. What are the most significant re-emergent biosecurity threats and do our current threat characteristics accommodate them?
96. Whilst attention is focused on scientists in an attempt to make them more aware of issues such as dual use, what efforts are being made to engage those who support industry such as investors and insurance companies?
97. How much biosecurity is too much?
98. What stops biosecurity information (i.e. from hack labs) being used for discriminatory purposes? (What is the control of the data?)
99. What are the considerations when a pesticide or chemical agent (or gene drive) is applied to an organism which has its range across another country with different rules?
100. Are measures to counter biosecurity and cybersecurity threats linked in the UK Government security strategy?
101. How can biosecurity topics be introduced to social science curriculums as those concerning nuclear threats had been during the cold war?
102. Do research centres merely provide the UK Government with forecasts and evidence regarding biosecurity threats, or help develop security policy? If the latter, what sort of platform can keep researchers and security experts systematically linked?
103. Are risks of bioterrorism as high as those of undeliberate biosecurity threats?
104. Can public awareness of indicative signs of bioterrorism be integrated in the UK Government campaign to raise awareness regarding more common types of terrorism?
105. Are countermeasures to bioterrorism geared primarily to respond to material instances or to pre-empt possible threats based on actionable intelligence?
106. What methods are employed to detect and verify actionable threats to biosecurity?
107. How do the American and European strategies to bioterrorism compare, and can the UK Government strategy integrate itself in both?
108. What are the obstacles to sharing intelligence and law enforcement resources regarding bioterrorism between various domestic and international agencies?
109. What role does the social media play in contributing to or concealing an act of bioterrorism?
110. Do fake biosecurity risks exist, and are they a problem?
111. Are we overestimating biosecurity risks and threats?
112. Do any current or near-horizon approaches towards broad-spectrum antiviral treatments (https://www.ncbi.nlm.nih.gov/pmc/articles/PMC4440912/) show significant or under-recognised promise for preventing or mitigating emerging viral threats in humans and animals?
113. How much harm is posed by the current budgetary challenges faced by the BWC Convention in terms of the effective functioning of the BWC?
114. How adequate is funding for major institutions playing a role in identifying and mitigating global biological threats (e.g. the World Health Organisation)?
115. What are the key challenges that Brexit will pose for UK biosecurity, and how can they be mitigated?
116. What specific disease threats are most exacerbated by the growing anti-vaccine movement in the UK, and how can these be mitigated?
117. How is the policy space structured by biosecurity better served by taking it out of the security framing?
118. How is improving one group’s understanding of biosecurity diminishing another’s?
119. How do we deal with the unknown knowns in biosecurity? (Unknown knowns are the things we don’t realise that another group knows).
120. What are the strengths and weaknesses of the different horizon scanning activities used by academics, the WHO and government agencies?
121. Have biosecurity horizon scanning activities been successful? That is, what is the rate of successful predictions and have they catalysed policy change?
122. What are the most appropriate horizon scanning tools (superforecasting, Delphi, prediction markets) for forecasting biosecurity issues in the UK?
123. What reforms would be needed to make the BWC effective (such as the addition of an enforcement protocol) and can the UK help push for these internationally?
124. Should there be a ‘review of review conferences’ under the BWC? That is, have review conferences been effective or catalysed any changes?
125. What are the most effective interventions that the UK can take to mitigate the Dark Web becoming a technological vector for bioengineering threats?
126. How often do biosecurity projects with private funding produce results that benefit the commercial interests of the financers?
127. What is the current composition (commercial, philanthropic, government and academic split) of funding in biosecurity and bioengineering in the UK?
128. Does the UK’s 2018 Biosecurity Strategy correspond to the responsible innovation principles of being responsive, reflective, anticipatory and inclusive?
129. Forecasting techniques have been shown to be ineffective at foreseeing ‘black-swan’ (low-probability and high-impact) events (Goodwin and Wright, 2010). However, making ‘anti-fragile’ systems and using devils advocates in forecasting tools could help remedy this deficiency. Have either of these approaches be used in forecasting activities for UK biosecurity?
130. How do we build an ‘anti-fragile’ (a system that gains from shocks and disorder) biosecurity system in the UK?
131. How can those with a public platform, e.g. academics, journalists or politicians, best advocate for improving biosecurity?
132. How can we best build capacity in Parliament to hold the government accountable for implementation of the UK National Biological Security Strategy?
133. In the face of lack of, or at best variable compliance with International Health Regulations, is there potential in a web-based platform to develop, strengthen and maintain relevant public health capacities in poorer countries?
134. What can be done to improve biosecurity in laboratories, particularly in terms of decreasing both accidental and intentional release rates?
135. Who needs to be involved in decision-making on governance measures for biosecurity in the UK?

1. The genus of the flowering plant family Convolvulaceae, commonly known as the bindweed family. [↑](#footnote-ref-1)
